# Community health worker payment processes: a qualitative assessment of experiences in two Indian states

**DOI:** 10.1093/heapol/czaf010

**Published:** 2025-02-15

**Authors:** Kheya Melo Furtado, Abha Mehndiratta, Sebastian Bauhoff, Swapna Pawar, Amy Luo, Anushree Jha, Margaret McConnell

**Affiliations:** Goa Institute of Management, Poriem, Sattari, Goa 403505, India; Goa Institute of Management, Poriem, Sattari, Goa 403505, India; Harvard University T.H. Chan School of Public Health, Boston, MA 02115, United States; Goa Institute of Management, Poriem, Sattari, Goa 403505, India; Johns Hopkins Bloomberg School of Public Health, Baltimore, MD 21218, United States; Goa Institute of Management, Poriem, Sattari, Goa 403505, India; Harvard University T.H. Chan School of Public Health, Boston, MA 02115, United States

**Keywords:** community health workers, compensation, payment processes, payment delays, motivation, digital payment systems, ASHAs, India

## Abstract

Community health worker (CHWs) remuneration has received some attention in terms of the design of incentives, however, there is a lack of systematic data on the processes by which CHWs are paid. We aimed to study existing payment processes including the role of digitization and its effects on CHW experiences with receiving full and timely compensation, and identify barriers and facilitators to the payment process. We studied payment processes for the Accredited Social Health Activist (ASHA) in India in two states with varying levels of performance and payment systems and conducted 53 in-depth interviews and eight focus group discussions across three categories of respondents (37 ASHA workers, 46 supervisors, and 34 managers/health system leaders). The data was coded thematically using inductive and deductive coding methods organized around five steps of the payment process: (i) recording of work, (ii) claim submission, (iii) claim verification, (iv) claim processing, and (v) payment disbursement. We observed complex sub-processes within each stage of the payment process that adversely impacted payment timelines, CHW workload, and motivation, even where digital tools provide support. Local administrative initiative and positive organizational culture overcame these challenges to standardize and simplify processes for recording work, submitting claims, and maintaining adequate funds, facilitating timely payments. Complete digitization of disbursement through the public financial management system improved timeliness, transparency, and satisfaction among CHWs compared to earlier cash and cheque-based payments. The potential digitization of service delivery records for claim submission was met with mixed perceptions among CHWs and their supervisors. Our study contributes to the body of knowledge on CHW compensation by delineating the processes by which financial incentives are paid and offering insights for low and middle-income countries to improve the efficiency of payment systems

Key messages

- Community health workers (CHWs) in low and middle-income countries (LMICs) often face inefficient payment systems due to receiving performance-based incentives rather than regular salaries. Despite extensive literature on CHW compensation design, the actual payment processes have received minimal focus.
- Our research identifies complex sub-processes within each stage of the payment process that adversely affect payment timelines, health worker workload, and motivation.
- Implementing standardized policies for reporting work and digital solutions such as payment software and direct bank transfers can significantly improve payment efficiency. However, effective payment improvements also depend on robust fund management, a positive work culture, and capacity building for digital adoption in the preliminary stages of the payment process.
- Our study identifies opportunities and challenges in the payment process that can guide improvements in CHW payment systems and motivation in LMICs.

## Introduction

Community health workers (CHWs) play a central role in the delivery of primary care in many low- and middle-income countries (LMICs). In this capacity, they bridge the gap between the population and the health system by overcoming geographical and social barriers to accessing health services (Peretz et al. 2020). CHW programmes have evolved over many decades (USAID 2015), and several LMICs now have national programmes that recruit, train, and deploy CHWs to provide essential health services within communities (Anon, 2021; World Health Organization 2017b). There is growing evidence of the effectiveness of CHWs in providing preventive, promotive, and curative primary care (Cometto et al. 2018), and their role in advancing health systems toward universal health coverage in LMICs was globally recognised for the first time in the 72nd World Health Assembly in 2019 (World Health Organization 2019).

Despite the growing importance and scale of CHW programmes, countries often struggle with compensating CHWs to attract, motivate, and retain personnel (Colvin et al. 2021, Nkonki et al. 2011, Anon, 2021). Global guidelines emphasise that CHWs should be appropriately and fairly compensated through financial incentives, though many still work for no financial compensation (Cometto et al. 2018, Ballard et al. 2022). In comparison to other cadres of health workers, CHWs are particularly at risk of inefficient and inadequate payment systems because they often work part-time, are not on the civil service payroll, and may not draw regular salaries, but instead are paid with a combination of non-monetary incentives and performance-based incentives (Ballard et al. 2021, 2022, Colvin et al. 2021).

While there is substantial literature on designing and optimizing CHW incentive and remuneration structures (Bhattacharyya et al. 2001, Glenton et al. 2010, Singh et al. 2015, Colvin et al. 2021), there has been little attention given to the process by which CHWs are paid (McConnell et al. 2022). CHW compensation involves reporting and verifying activities at multiple administrative levels before payments are disbursed and reach the health worker. The complex processes preceding and following the payments can lead to inefficiencies such as ‘ghost’ workers, duplication of records, administrative burden, unauthorized deductions, and late payments, all of which can impact CHW motivation, performance, and retention (Heymann 2015, Bangura 2016, Joshi et al. 2020, Digital Financial Services and Health Working Group 2021, McConnell et al. 2022). Thus, understanding the processes involved in documentation of work, payment approvals, and disbursement is an important step in improving CHW payments, with implications for health system performance.

Digitizing payment processes has been put forward as a specific solution to increase the efficiency of payment systems by improving the timeliness of payments, decreasing leakages of resources, and reducing the time spent on processing and accessing these payments (mSTAR/Liberia, n.d.; Bangura 2016, Yehualashet et al. 2016). Digital processes could also create transparency for better monitoring and accountability in timely compensation, improving healthcare worker convenience and motivation (GAVI 2020, mSTAR/Liberia, n.d.). However, the ways in which digitization interacts with longstanding health system factors to influence payment processes needs further exploration.

Our study aims to address this knowledge gap by systematically describing existing payment processes for CHWs, i.e. the steps involved in tracking work, receiving approval, and disbursing payments. We explore the effects of methods adopted in these processes, including the role of digitization, in CHW experiences with receiving full and timely compensation. We aim to identify opportunities and challenges for improving payment systems and CHW experiences.

### Study context

We studied payment processes for the Accredited Social Health Activist (ASHA), a CHW programme within India’s National Health Mission. The ASHA is a ‘voluntary’ community health worker, usually serving the community to which she herself belongs. She is usually a married, divorced, or widowed woman with at least 12 years of education, although the latter criterion can be reduced according to need (Ministry of Health and Family Welfare, Government of India 2012). ASHAs are tasked with health promotion activities and enabling access to primary health care services. Each ASHA is intended to serve a population of 1000 individuals. However, these numbers range from 285 in Arunachal Pradesh to 1241 in Bihar, with variations across districts and states (National Health Systems Resource Centre 2022). India’s ASHA programme was introduced in 2005; there are currently ∼983 032 ASHAs who are meant to serve as the first point of contact of the public health system with the community, making it the largest community worker programme globally (National Health Systems Resource Centre 2022).

Payments to ASHAs are linked to performance for a comprehensive set of services, with unit payments defined for each activity. These include, maternal and child health, adolescent health, national programmes like the Revised National Tuberculosis Control Programme, National Leprosy Eradication Programme, National Vector Borne Disease Control Programme, Universal Non-Communicable Disease Screening, and those for maintaining safe drinking water and sanitation. Services related to maternal and child health constitute the major proportion of an ASHA’s daily workload. Based on the demographic distribution of the catchment population and volumes of services delivered, ASHAs typically receive a total incentive payment of between 36 to 50 United States Dollars (USD) per month (Ministry of Health and Family Welfare, Government of India 2018).

Indian states have evolved their ASHA payment systems over time. This includes moving from cash to cheque payments, and later to direct bank transfers. Other payment systems have combined performance-linked incentives with some amounts of fixed honorariums, and some have more recently implemented digital applications for claims processing. However, challenges with irregular and delayed payments persist, impacting motivation and leading to widespread frustration among ASHAs (Wang et al. 2012, Saprii et al. 2015, Singh et al. 2017, Bisht and Menon 2020, Pulagam and Satyanarayana 2021, Babu 2022, Sattar and Raman 2023).

## Methods

### Selection of ASHA activities and applicable payments for study

We selected activities related to antenatal care, institutional deliveries, and child immunization because the major portion of ASHA compensation relates to maternal and child health services. ASHAs receive compensation based on patients’ utilization of care. They receive ∼6 USD for every woman completing antenatal care, ∼3.6–4 USD for every institutional delivery, and ∼4.8 USD for the complete immunization of a child (see supplementary Table A1 in the online supplementary material for the full list of incentives for ASHAs related to maternal and child health services).

### Selection of states

We selected two large states, Maharashtra and Assam, for their contrasting characteristics, health system performance, and payment systems to better understand variation in payment processes, digital adoption, and their consequences.

Maharashtra (MH) has a population of 112.3 million and is located in western India. It is categorized as a ‘high performing state’ in terms of its performance on maternal health outcomes and reports a maternal mortality ratio of 33 per 100 000 live births (Government of India 2022). The state disburses payments digitally through the Public Financial Management System (PFMS), which directly deposits payments into the ASHA’s bank account. At the time of the study, the remainder of the payment process (i.e. recording of work and claim submission, verification, and processing) was manual. Maharashtra previously used claim processing software. About 18 months before the start of this study it was discontinued by the State Health Society for upgrading.

Assam (AS) has a population of 31.2 million and is located in the north-eastern region of India. It is categorized as a ‘low performing state’ with a maternal mortality ratio of 195 per 100 000 live births (Government of India 2022). Assam has digitized ASHA claim processing and disburses payments through the PFMS into ASHA bank accounts.

These differences in the states presented an opportunity to study a broad spectrum of facilitators and barriers in the payment process.

### Selection of districts and health centres

We sought to select districts in each state that represented ‘typical’ performance—neither best nor worst performing districts. For both states, we extracted district-level data from the most recent (2019–21) round of the National Health Family Survey-5, India’s Demographic and Health Survey, on three key indicators linked to the activities included in our study, i.e. the proportion of mothers who had at least four antenatal care visits, the proportion of institutional births among all deliveries, and the proportion of children aged 12–23 months who were fully vaccinated.

We calculated summed scaled values of the three indicators and arranged districts in each state into performance quartiles. In each state, we selected one district each from the second and third performance quartiles, respectively. Within each selected district, we selected one health administrative block within a 3-h travel distance of the district capital (excluding the capital). Since ASHAs are affiliated with primary health centres (PHCs), we randomly selected one or two PHCs within the block to obtain a sampling frame of at least 15 ASHAs from which to randomly select for inclusion in the focus groups and interviews.

### Data collection and analysis

We collected data from July to November 2021 from three groups of respondents to understand the complete payment process for ASHA workers. The first group consisted of 37 ASHA workers, selected randomly from the complete list of ASHAs assigned to the identified PHCs. The second group consisted of 46 ASHA supervisors and auxiliary nurse midwives (ANMs), selected randomly from the complete list of supervisors and ANMs assigned to these PHCs. ASHA supervisors are the immediate supervisors of ASHAs in both states, while ANMs work closely with ASHAs and verify their work. The third group consisted of 34 managers and health system leaders at the PHC, block, district, and state levels who were part of the payment and supervision structure for ASHAs. We conducted 53 in-depth interviews (IDIs) and eight focus group discussions (FGDs), with eight members per FGD across the three categories of respondents. A complete list of respondents, organized by interviews and focus groups, is provided in supplementary Table A2, see online supplementary material.

**Figure 1.**
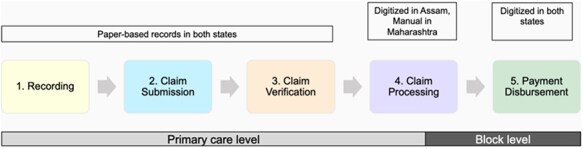
ASHA payment process steps.

We developed separate semi-structured interview guides for each respondent group. We conducted FGDs with ASHAs and their supervisors to elucidate the details of the steps in the payment process, which we categorized into recording of work, claim submission, claim verification, claim processing, and payment disbursement (Fig. 1). The FGDs and interviews were conducted in Hindi in Maharashtra, and in Hindi and Assamese in Assam. Interviews for the third group—managers and leaders—were conducted in English. The first authors conducted all interviews in Hindi and English; for Assamese, an interpreter supported the process. All interviews and FGDs were conducted in person, except for three interviews which were conducted by video call (two in Maharashtra and one in Assam). All IDIs and FGDs were audio-recorded after obtaining consent from participants.

We transcribed all audio recordings, translated them into English and checked for accuracy and consistency with the original language. We carried out a thematic analysis of the transcripts initially using deductive coding. We developed a list of parent codes based on our categorization of the payment process steps, and sub-codes based on intermediate goals of health financing policy, i.e. efficiency, transparency, and accountability of the payment process (Kutzin 2008). We inductively included worker motivation as a sub-code and identified and reported additional common themes (see supplementary Table A3 in the online supplementary material). The first authors used the code list to code the first transcript and finalize the codes. Two co-authors coded transcripts following a standardization process using the first transcript. Periodic team meetings were held during the coding process to discuss and clarify subjective considerations in the data and ensure a consistent approach. The first authors conducted further quality checks of coded data. The data analysis was supported by NVivo version 12 software and thematic analysis of the coded data was carried out.

### Ethics approval and consent

The study was reviewed and approved by the Institutional Review Board and the Board of Research Ethics at the authors’ institutes. We obtained permission to conduct the study and recruit participants from authorities of the National Health Mission programme of both states, the overarching scheme of the Ministry of Health and Family Welfare that implements the ASHA programme. Written informed consent was obtained from all participants before conducting interviews and FGDs.

## Results

**Figure 2.**
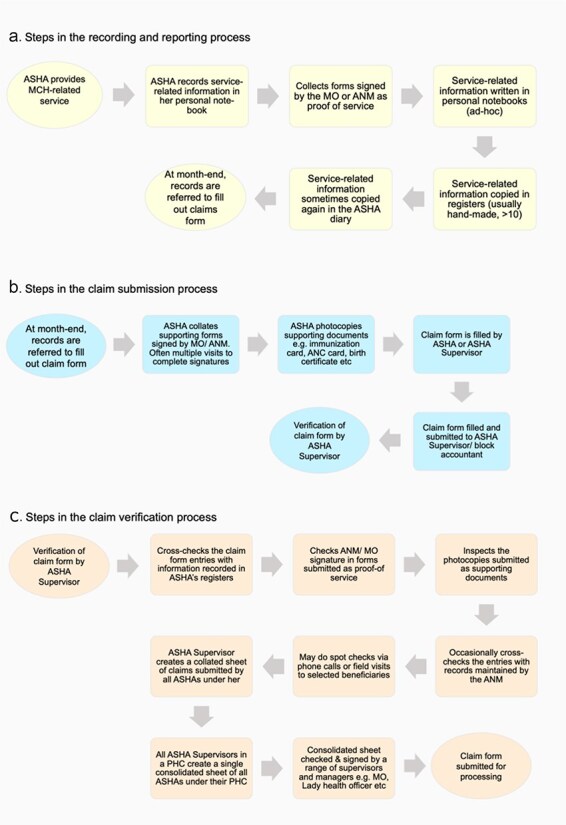

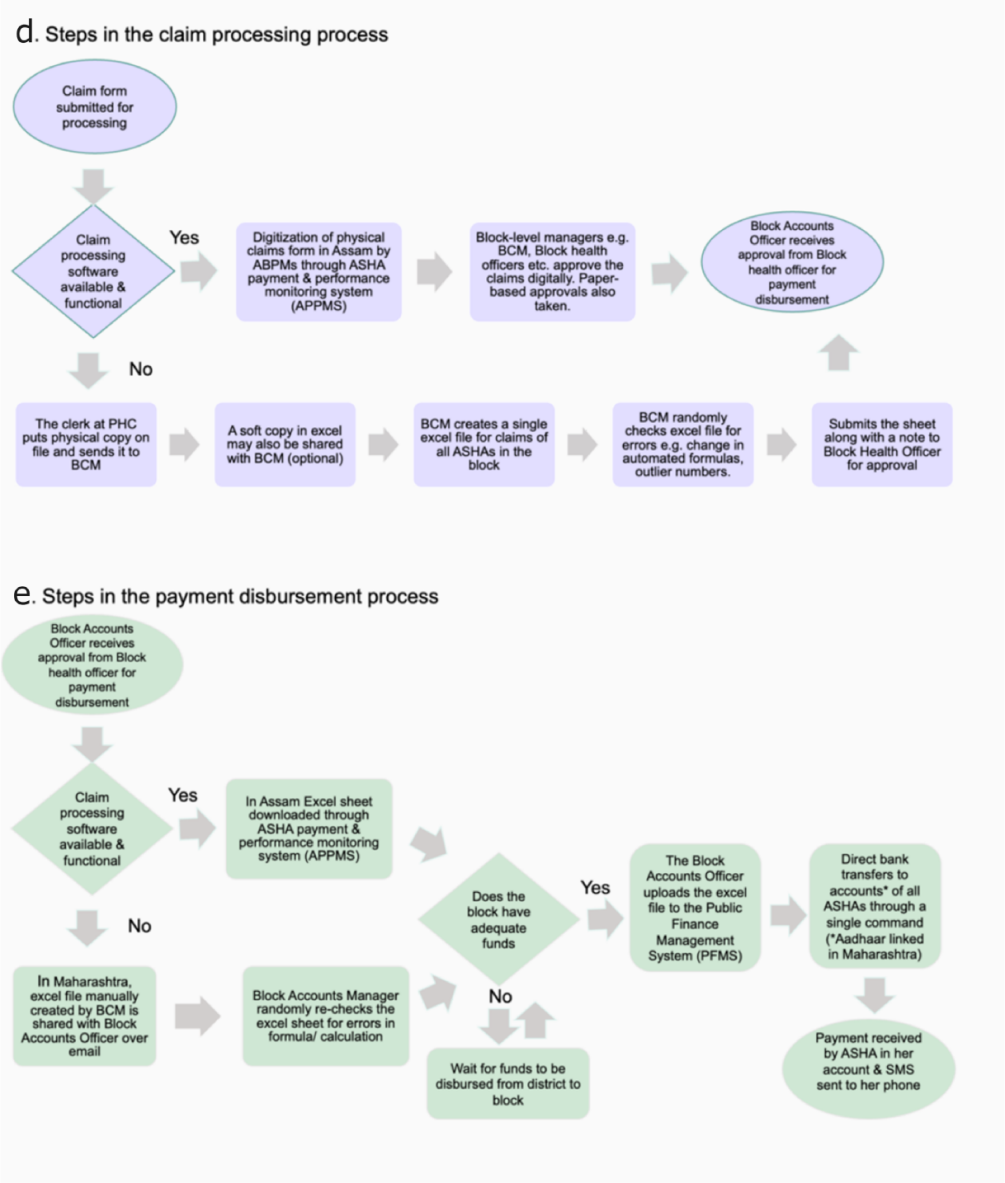
Sub-steps within each stage of the ASHA payment process: (A) the recording and reporting process; (B) the claim submission process; (C) the claim verification process; (D) the claim processing process; and (E) the payment disbursement process.

We found that ASHA payments involved an elaborate set of sub-steps in each of the stages of the payment process (Fig. 2). We describe the insights gained from studying these processes in the subsequent sections, organizing our main findings around each main step of the payment process flow: i.e. (i) recording of work; (ii) claim submission; (iii) claim verification; (iv) claim processing; and (v) payment disbursement. We also extracted the linkages between payment systems and the tools they provided for monitoring and supervision of ASHA performance and payment timelines. A summary of the facilitators and barriers to the payment process is described in Table 1.

**Table 1.** Summary of barriers and facilitators in the ASHA payment process

Payment process step	Barriers	Facilitators	Role of digitization
1. Recording	Lack of standardized and explicit policies leads to excessive documentation and increased workload Manual paper-based recording system creates inefficiencies	Clear written policies that define the roles and responsibilities of various stakeholders relevant to the payment system, with information on required documentation and timelines for each step, reduces duplicative practices and out-of-pocket expenses for ASHAs Standardization of processes by local supervisors and managers, with staff training, simplifies the process and reduces payment delays and out-of-pocket expenses	Both states have manual processes for the first three steps of the payment process. There was agreement that digital tools could help reduce paperwork related to service delivery records and timelines for submission of claims, but also apprehension about the adoption of these tools within current technical and infrastructure capacity constraints in certain areas
2. Claim submission	Ambiguity on mandated documents for claim submission pressurizes ASHAs to provide inordinate proof of work done, causes out-of-pocket expenses for ASHAs, claim deductions, and payment delays		
3. Claim verification	Verification process mainly dependant on record review, which gives impetus to a paper-based bureaucracy	Process re-design in consultation with stakeholders can simplify verification process	
4. Claim processing	Risk of errors and need for multiple checks where claim processing is manual Delays in digitizing claim forms	Digitization of claims minimizes errors, need for multiple checks, and reduces payment timeline Organizational culture supports timely payments even in the absence of digitization	Digitization of claim processing in Assam minimizes errors and the need for multiple checks; however, payments are timely only when teams prioritize the digitization of these forms for further approvals. The use of software had been paused in Maharashtra at the time of the study.
5. Payment disbursement	Delayed payments where districts are unable to retain sufficient funds for timely transfers to blocks.	Efficient accounting practices that result in regular fund flow The PFMS enables an integrated payment process Money received directly into bank accounts is preferred by ASHAs over earlier systems of cash and cheque-based payments Reports generated through the software when used, support monitoring and supervision of ASHA performance and payment timelines	Both states benefit from the use of the PFMS that creates an efficient system of disbursement of payments to all ASHAs at the same time, directly into their bank accounts. These methods greatly reduced payment timelines and increased ASHA satisfaction as compared to earlier systems Assam benefitted from the use of the software which generated useful reports for supervising ASHA performance, providing feedback, and monitoring payment timelines

### Recording of work

ASHAs reported many steps (often duplicative) required to document and submit claims (documented in Fig. 2A). There was also substantial variation in official processes across states and local jurisdictions.

#### Excessive documentation and lack of clarity on policies increase workload

ASHAs reported a significant workload burden due to excessive documentation and a lack of clear policies for recording work. In most places, an individual ASHA maintains more than ten different registers for service-related information, with each register typically corresponding to a specific category of incentives. It is common for ASHAs to carry their paperwork home, often transforming their living spaces into makeshift offices. There are significant variations in the number, type, and content of registers ASHAs maintain, as well as in the supporting documents required for the payment of claims. ASHAs expressed that maintaining multiple records for the same service was demotivating. The ASHA supervisors acknowledged that the extensive documentation burden not only impacted an ASHA’s productivity but also carried an opportunity cost, as the time spent on paperwork limited the time available for providing essential health services. They emphasized the need to simplify the ASHAs’ paperwork to allow them to focus more on actual service delivery.

‘*Yes, in writing records, I feel very low. Yes, again and again* (sic)*. Right now, this pregnancy record is written here, and it’s written there too, then about the child’s record same, it’s written here and there. The entry of a single name has to be done in five registers*.’ (MH ASHA 1)

‘*If the system is made a little less cumbersome for them, they will be happier. Half of the time goes in the documentation part… maintain this, maintain that… if it is made simpler, they can focus on their work which is being in the community and paying visits*.’ (MH Health System Leader 16)

Despite Assam having a state-level explicit policy on which records ASHAs needed to maintain and submit to receive their incentives, in one district in Assam, most local stakeholders were unaware of this policy. Maharashtra did not have a state policy regarding ASHA documentation at the time, but one of the districts innovated and developed a local policy to systematize the ASHA documentation and claim submission process.

#### Mixed perceptions toward a future transition from the current paper-based process of documenting work to a digitized system

In both states, most ASHAs agreed that digitization would simplify the documentation at the point of service by reducing paperwork, making work less time-consuming, giving access to complete data, and reducing the need to carry heavy physical records. Supervisors also concurred that digital tools would make it easier to monitor the status of their work; however, some respondents noted potential challenges for those who are less educated: ‘*operating on mobile is not possible for everyone*’, and expressed concerns about access to smartphones or tablets and internet connectivity: ‘*we have a lot of network problems here in the village. If we have to search for the network, we have to come five or six kilometers ahead… That’s why we feel that we don’t want that mobile*.’ (MH ASHA 12). Supervisors in areas where ASHAs were less comfortable with the general use of mobile phones were specifically apprehensive about the implications for themselves in facilitating the adoption of these tools, as this would increase their own workload. ASHAs emphasised that the state ‘*must provide mobile* (phones) *because whenever we go to the village, for an ANC, for a child visit, or for some HBNC (home-based neonatal care) we have to carry all this with us. It is very difficult, so we don’t have to carry so much if we get mobiles, we have to walk all the way in the sunlight, and in rain it all becomes wet…*’ (MH ASHA 1). There was a broad consensus, however, that if the health department provides access to smartphones/tablets, and gives training to ASHAs, they can learn to use mobile and software tools:

‘*If training is given, mobile can be an option because they have a lot of paperwork. And if she feeds the data online, then we can see from here also. And their paperwork will also be reduced. So mobile is a good option but all ASHA will have to be trained for it*.’ (AS Health System Leader 7)

### Claim submission

At the end of the month, the ASHA fills out a claim form with a summary of all the work done in the preceding month and submits this along with multiple signed documentation as proof of service to her supervisor (Fig. 2B).

#### Laborious processes for submission lead to out-of-pocket costs for ASHAs and payment delays

In all four districts, ASHAs submitted supporting evidence in the form of photocopied medical and personal records of the mother and child, along with the claim form: ‘*They have different papers for each incentive; each one has to be signed. Then they have to Xerox [photocopy] so many papers*.’ (MH Health System Leader 17). The type and number of photocopies submitted as proof varied across the four districts. These photocopies caused substantial out-of-pocket expenses: ‘*In Xerox, we spend Rs.150–200 per month for the proof we give them*’ (MH ASHA 5). The expense is ∼5% of the average monthly payment ASHAs receive. ASHAs recounted that the collection, photocopying, and returning of records to patients was burdensome. In many cases, ASHAs were required to travel long distances between patients’ homes to obtain records or to faraway shops to make photocopies.

In one district of both states, many stakeholders lacked clarity on documentation for claim submission, and ASHA payment schedules were delayed. On the other hand, in the other two districts that had clear guidelines on claim submission and timelines, respondents indicated that payments were timely. Furthermore, ASHAs, supervisors, and managers noted that the number of claims rejected due to incomplete documentation and the amount of excessive and duplicative documentation had decreased. Assam has developed a standardized claim form (Master Claim Form), which the ASHA uses to summarize her monthly work to claim financial incentives. This form has a comprehensive list of eligible services and related incentive amounts that the ASHA can reference at the time of claim submission. The introduction of the form has streamlined the claim submission in comparison to the past: ‘*(with) one Master Claim Form I think we have saved 30–40 Xerox pages*’ (AS Health System Leader 3). In one Assam district stakeholders were aware: ‘*There is a guideline by the state government, which clearly indicates the documents required for incentives*’ (AS Health System Leader 14). There are, however, delays in updating and distributing the Master Claim Form from the state level when the incentive policy changes, and few stakeholders in the second Assam district were aware of the state policy.

In Maharashtra, both study districts had locally developed claim forms and relied on nationally published documents like the ASHA diary for information on incentive types and amounts. The ASHA diary was, however, not used much for its primary purpose of recording and reporting in any of the districts studied due to its small font size, limited available recording space, and delays in printing and supply from central stores. One Maharashtra district streamlined its local policy substantially to address audit queries, payment delays, and out-of-pocket expenditure incurred by ASHAs: ‘*…we had a meeting with the CEO madam and in that we had a discussion about what should happen to us for the audit of ASHA. Then we made a copy of all these indicators, took its approval from the CEO madam and made a list of all those documents*’ (MH Health System Leader 13). This significantly reduced, though not entirely eliminated, the ASHA’s out-of-pocket expenses due to the replacement of mandated photocopies with supervisor signatures for several incentives: ‘*…ASHA can’t afford to pay so much for all that number of Xerox for just one case... so we have prepared a format, where all the heads are written and under each head, they have to write every month how much work has been done under that head and how much incentive they are going to get for that work…*’ (MH Health System Leader 14).

### Claim verification

ASHA supervisors verify claim forms and cross-check information using multiple documentary sources, and in some cases through field-based verifications of service delivery (Fig. 2C).

#### Verification mainly depends on a review of signed records, which creates a bureaucracy rather than a robust authentication mechanism

Verification of claims is dependent on the review of mandated signatures on records. There was limited application of other verification methods like stakeholder interviews. Document verification focused mainly on checking the completeness of records to safeguard against a future audit rather than reviewing whether these were accurate. Signatures from multiple supervisors and managers were necessary for verification before and after ASHAs submitted their claim forms for processing. The number and type of signatories required were not uniform across the districts. These signatures increased the ASHA’s workload, requiring ASHAs to commute between multiple sites for the mandated signatures. At times, she had to travel long distances to referral hospitals and beneficiary homes at her own expense to obtain the required signatures: ‘*So, to take her sign, ASHA has to go to the sub-centre, which is often far away. Now our sub-centre is far away from us, so if she has to go two or three times, then it becomes difficult for her. Because there is a ticket cost for coming and going*’ (MH Health System Supervisor 17). The need for numerous signatures from multiple stakeholders has led to a paper-based bureaucracy creating delays, and was often a rate-limiting step in the payment process. ASHAs and their supervisors strongly argued for simplification of the system: ‘*there should be only one or two signing authorities from whom she should take the signatures*’ (MH Health System Supervisor 3), and ‘*… they should give the signature immediately. ASHA should not go four or five times to get the signature done*.’ (MH Health System Supervisor 2)

In one district in Maharashtra, local initiatives led to the simplification of the verification process to curtail redundant signatures and reduced the burden on ASHAs:

‘*…there was a lot of chaos, as programmes used to come, new rules were drawn up, sometimes they used to say that we need the signature of Gramsevak, sometimes they needed signature of Sarpanch, there was no such uniformity at all. So, we reduced those things… It also reduced the paperwork; ASHA’s confusion was also reduced, and it became easier for us to check*.’ (MH Health System Leader 15)

They decreased the number of signatories and mandated supporting documents and photocopies: ‘*…We thought that instead of maintaining so many documents, if all the details come in a single paper and were commonly signed then it would be easier to verify as well… paper waste will also not be much. So, we created it ourselves…*’ (MHHealth System Leader 15)

### Claim processing

In Maharashtra, claims processing was a manual process requiring several sub-steps. In Assam, claims processing was digitized through the ASHA Payment & Performance Monitoring System (APPMS) (Fig. 2D).

#### Errors and the need for multiple checks in the manual process are reduced where claim processing is digitized, reducing payment timelines

The manual claims process observed in Maharashtra was prone to human error and duplication of work since multiple stakeholders needed to compile the data by hand. Errors in the selection of payment codes and spreadsheet-fed formulae could occur and a substantial amount of time was spent manually checking for these errors, precipitating further delays in payment. As one manager explained: *‘Sometimes what happens is that while entering the data the formula itself gets deleted by them, if the excel is not locked… So, if it comes to the block, then we have to check all to see what mistake they have done. Sometimes if the formula is wrong, the payment becomes less and more (sic)*’ (MH Health System Leader 8). This risk and fear of inadvertent errors creates an additional workload and stress for the managers responsible for compiling final payments, especially when managing larger teams of ASHAs and consequently being accountable for disbursing large sums of money.

In Assam, where the payment software was available, respondents indicated that it reduced the need for multiple manual checks of data for errors. The software enabled the generation of a digital form based on the physical claim submitted by the ASHAs after their supervisors had verified it. Once the data was entered into the software, the digitized information was used for obtaining further necessary approvals and uploaded on the PFMS for disbursing payments. In one district, this digitized step had improved overall payment timelines as compared to the earlier manual compilation of data ‘*Earlier, when… it used to be made in the Excel sheet, payment was done in 2 to 3 months. We do not have any backlog since this software has come*.’ (AS Health System Leader 11)

#### Organizational culture supports timely payments in the absence of digitization and delays payments even where digital tools are available

Despite manual processing in the absence of digital tools, one district in Maharashtra was able to ensure timely payments to their ASHAs. Multiple stakeholders in this district emphasised their responsibility toward prioritizing ASHA payments among their many tasks, and ensured that the necessary approvals were sought in time, enabling timely payments. As mentioned earlier, the district had also proactively initiated a standardized format and process for claim submissions and verification. This district showed high levels of initiative and motivation in ensuring ASHA payments were timely. As one leader explained,

‘*The coordination here is very good, communication among each other... Knowing when to give priority to which thing. We also have a little emotional corner for ASHAs. We feel that any way they do not get much, there are many ASHA who also have loans. So we have to do that with priority. So, I and BCM [Block Community Mobilizer] work accordingly to see how we can get the money deposited in Asha’s account on the first [day of the month]*.’ (MH Health System Leader 15)

On the other hand, despite the digitization of the claim form, the transfer of information from paper to digital form was a bottleneck in one district of Assam, which experienced delays in inputting digitized claims for further processing of payments. ASHAs and their supervisors consistently reported that payments were delayed in this district: ‘*We won’t get (payments) in every month… it comes within or after 2 months it is always late*’ (AS ASHA 1). As reasons for the delays, managers cited challenges such as being assigned multiple tasks and competing priorities, which prevented them from completing this payment process step in time. They also implied that timely payments were not a top priority within their broader work:

‘*…there is no delay, see there is some technical problem and that ASHAs will never understand… we also have to earn a salary, have to do other work too. Those people understand that we will continue to pay ASHA only, it doesn’t happen like that, we get very little time (sic). No benefit will be there by explaining to ASHA, they will not understand… there is only not the issue of the money only. Then later the payment is done and there are some holidays too, …banks are also closed*.’ (AS Health System Leader 17)

### Payment disbursement

Once claims were processed and approved for payment, the disbursement step was digitized in both states through the PFMS, an application for tracking government (central and state) fund flows to disburse money from administrative blocks (sub-district unit) to ASHA bank accounts (Fig. 2E).

#### The PFMS enables all health workers in the administrative area to receive payments at the same time, with fewer possibilities of errors compared to earlier methods

Accounts managers use the PFMS and digital signatures to transfer funds to ASHAs’ bank accounts, though physically signed paperwork is sometimes retained as backup and to reinforce accountability.

The process of linking ASHA bank accounts to the PFMS application has significantly simplified the process of disbursement. This direct deposit system integrates all bank accounts with the application in a one-time process, such that only health worker names and corresponding payment amounts need to be verified by the manager each month. As managers indicated,

*‘This PFMS system is much better as compared to earlier RTGS [Real Time Gross Settlement, a means of transferring funds from one bank account to another]. Because in RTGS we had to check her account details every time. Had to check her Indian Financial System Code (IFSC) code, and bank details. Now once it is saved in the system, then we just have to look at her name and enter the payment, there is no need to check the account details again. …And since there was more clerical work in RTGS, there could be mistakes in it*.’ (MH Health System Leader 5)

#### Money received directly into bank accounts is preferred by ASHAs over earlier systems of cash and cheque-based payments, improving satisfaction with the payment system

Before the creation of bank accounts for payment purposes, ASHAs received cheques or cash payments. The earlier methods were operationally more time-consuming and error-prone in terms of payment amounts disbursed. Many of the ASHAs opened bank accounts only upon being appointed and were pleased with this mechanism: ‘*We love when it comes to the account because then we have savings…*’ (AS ASHA 11). The managers explained what this form of financial inclusion meant to them, ‘*…they have opened* (accounts) *especially for this… because they do the work of ASHA, because of that these women could come ahead a little. As it used to happen earlier, everything was seen by their husbands only (sic). She was only confined to looking after household chores and taking care of children*. (MH Health System Leader 15)

Accessing funds was quicker, as there was no need to physically travel to collect their payments or deposit cheques into banks. ASHAs received text message notifications when payments were made. State and district managers indicated that possible unfair and corrupt practices related to cash payments that may have earlier resulted in ASHAs receiving less than the full incentive payments have now been curtailed. In Maharashtra, these bank accounts were also linked to the unique national identification number of ASHAs (Aadhaar). This further ensures that payments are made to the rightful beneficiary.

#### Payments are delayed where districts are unable to retain sufficient funds for timely transfers to blocks

To ensure regular payments to ASHAs, the timely transfer of programme funds from the central government to states, and its further allocation down to districts and blocks, is crucial. Maintaining sufficient funds at the district level was essential for timely disbursements at the block level. District and block officials in both states prioritized ASHA payments once funds were available, initially allocating cash transfers to primary healthcare beneficiaries before covering ASHA remunerations. However, occasional fund shortages at the block level often stemmed from issues in managing transfers and retaining liquidity. ASHAs were often provided this explanation as the reason for the delay of their payments, ‘*…when we ask our block facilitator (supervisor), they tell us that the money has not come at the district level. If it comes from there, then only will we be able to distribute (money) here (block)*’ (MH ASHA 1). Some districts appeared to be able to do this better than others, based on the ability of the accounts managers to appropriately manage their National Health Mission funds (the overarching programme that provides the requisite budget for ASHA payments). This required independent initiative in re-appropriating budget allocations: ‘*whatever happens, we first give it from the available balance, and even if the balance does not come from the state, then also we transfer from the interest itself. Or else whatever interest they (block level) have, or the balance is available with them, under any head—means additionality, Reproductive and Child Health (RCH) , whatever is there, they are given a letter that you have to pay… ASHA from the same* (MH Health System Leader 12). Support provided by senior managers in such districts seemed critical to enable these actions.

### Monitoring and supervision of payment process

Both states reported monitoring the payment processes through monthly team meetings. We found that the digital application was specifically useful to generate reports that could be used to ensure timely payments, as well as to provide supportive supervision and performance monitoring.

#### Reports generated through the digital application support monitoring and supervision of ASHA performance, payment timelines, and the provision of specific feedback

In Assam, information on an ASHA’s individual performance (at the level of each activity assigned) was facilitated by the payment software available to senior management. Managers at the block, district, and state levels could simultaneously view reports and use these for recognition and awards.

‘*Earlier Government of India used to nominate the best performing ASHA, for us also it was very difficult to know how to nominate the best performing ASHA. Now at least we have been told that ASHA payment is one of the systems, their average monthly income, or if family planning division has claimed that please nominate for one of the best performers (sic). So, we will filter the family planning activities; if some RCH, some maternal health division we will filter the maternal health activity then automatically the system will say this Asha is claiming more than the national, state average (sic)*.’ (AS Health System Leader 3)

Similarly, the reports could be used to provide feedback to ASHAs, allowing them to increase specific activities and their overall earnings:

‘*Now if we look at the graph, then earlier it (compensation) was 3000−4000* (rupees)*, 3000–2000 like this. Because those people (ASHAs) were not aware, we were not able to guide those people (sic). Therefore, it was less. Because those people were not able to give feedback by talking to us verbally. Which activities they are doing well and which they are not (sic). When we are analyzing, we can specify, that you are not doing this claim. Why are you not doing it? What is the problem? Why is it not happening? So gradually and block-wise also we can see that if they have done 2000–3000 in any of our blocks, and if someone does 5000 then what is the problem? By identifying all this, we can tell those people that you improve yourself (sic)*.’ (AS Health System Leader 6)

Simultaneously, these reports had also been used for disciplinary actions, including replacing inactive ASHAs, enabling effective oversight.

The lack of digitized payment information in Maharashtra made access to actionable information cumbersome due to the need to manually generate these data. For reporting purposes, block functionaries faced challenges in providing detailed payment records for ASHAs over a longer period, as this had to be manually compiled from several sources.

‘*So due to different payments, we did not have much information, so can’t confirm about the payments made to Asha is correct or not. If the software was there at that time, then they would not have asked for the report from us, they could have downloaded the month-wise, year-wise data all themselves sitting at the state level. We could convert 4 years, even 5 years of data in Excel with the software*’. (MH Health System Leader 8)

The availability of the reports at the district level in Assam also provided managers with a tool to monitor compliance to timelines for each phase of the payment process and address delays.

‘*The time which has been given to supervisor she has to complete within that time frame. The time which has been given to ABPM (additional block programme manager), has to be completed in that time. If something happens, we come to know from the report. That they have not done as per the timelines of this month (sic). So that we can bring all of them on track. Because our report is with us. Everybody is alert that I have to complete the report within my timelines*.’ (AS Health System Leader 6)

Managers can be held accountable for payment processing delays, based on this information. However, these tools were used in only one of the two districts studied, highlighting that access to digital tools does not necessarily ensure their uptake.

A summary of the ASHA payment processing facilitators and barriers is given in Table 1.

## Discussion

We aimed to study CHW payment processes to identify the challenges and opportunities for improving the payment systems and CHW experiences. A significant challenge was the reliance on laborious, paper-based methods in the first three steps of the process, i.e. recording of work, claim submission, and verification, a practice that was often de-motivating and affected payment timelines adversely, due to the lack of standardization, clear policies, or clear communication. We identified, however, a mixed perception regarding the potential of transitioning these steps to a digitized system to reduce paperwork and standardize processes, where some expressed concerns over the use of smartphones or tablets, particularly in areas with poor internet connectivity. Supervisors were also concerned about the added workload of training CHWs to use digital tools, especially those who were technologically less proficient. Real-time digitization of records at the point of care delivery, if introduced, is likely to be more efficient even for payment systems, but strong capacity building and the IT infrastructure are prerequisites for its effective deployment (Feroz et al. 2021, Singh et al. 2021, Borges Do Nascimento et al. 2023). Solutions that link existing digital applications used by ANMs (Government of India, n.d.) (who work closely with ASHAs) or health facility records to the ASHA payment applications, could be explored as a means to input partial data on service delivery and also serve as verification in the ASHA payment process. There is, however, a risk that the dependence on paper-based records may persist even after the introduction of digital tools, as observed in our study and in other settings that retain a co-existing system of paper and electronic records, in case the electronic system fails (Agoro et al. 2018).

Digitization of the final stages in the payment process—claim processing and payment disbursement—positively impacted payment timelines when paired with effective local governance. This aligns with beneficial outcomes from similar software in other Indian states (Joshi et al. 2020) and supports LMIC evidence that digital payments increase efficiency, reduce leakages, and save processing time (Bangura 2016, Yehualashet et al. 2016, mSTAR/Liberia, n.d.). Additionally, digital payments have been seen to improve transparency, accountability, and healthcare worker motivation (GAVI 2020, mSTAR/Liberia, n.d.). Our study elucidates how these benefits are mediated by the characteristics of the payment process steps. Digitization had simplified the disbursement process through the integration of CHW bank accounts, and reduced time, errors, and corruption. Direct bank transfers were unanimously considered by CHWs to be superior to earlier systems of cash and cheque-based payments. Many of them had opened bank accounts for the first time for this purpose and enjoyed greater financial independence, savings, and access to bank loans. The deep penetration of Indian banks and access to mobile technology in rural India (Dahiya and Kumar 2020) have been key enablers in this transition. ASHAs and managers had better access to payment-related information through text-message alerts, bank statements, and PFMS records, avoiding possible leakages and improving the completeness of payments received. The generation of reports through digital systems improved accountability as these were used to monitor payment amounts and CHW activities, enabling recognition, feedback, and even disciplinary actions when necessary. Our observations thus contribute evidence from the health sector that aligns with global experience on the role of digital financial transactions in transforming payment systems for other development sectors within LMICs (mSTAR/Liberia, n.d.; Young 2013, Braniff and Sotiriou 2018, Breza et al. 2020, Blumenstock et al. 2023).

We also observed that digital solutions were effective only when supported by the following key health system factors. First, the availability of funds at the district level was crucial for timely payment disbursement, and some districts faced challenges in this regard. Good accounting practices at all administrative levels were key to maintaining cash flow, which directly impacted the ability of the system to disburse payments on time. Complex public financial management practices have been previously reported (Choudhury et al. 2017, World Health Organization 2017a, Wishnia and Goudge 2021), and efforts to simplify these are essential for ensuring timely CHW payments. Solutions such as adopting a single bank account for all health-related expenditures across administrative levels are being piloted in some states and have the potential to alleviate these challenges. Second, a positive organizational culture among managers and coordinated team efforts to prioritize CHW payments each month is necessary. Third, setting and ensuring compliance with clear written policies that define the roles and responsibilities of various stakeholders relevant to the payment system, with stipulated timelines for prescribed processes and steps, standardization of processes, and associated training of staff is required. These factors directly curtailed the practice of excessive documentation, reduced the rate of claims rejected due to incomplete documentation, minimized out-of-pocket expenditure toward unnecessary photocopies, and expedited payment timelines. Such persisting pain points for CHWs and their supervisors as also observed in earlier studies (Wang et al. 2012, Singh et al. 2015, JSI 2017, Ormel et al. 2019, Wahid et al. 2020) can thus be avoided.

Our study faced several challenges and limitations. We observed a noticeable hesitation among some health system leaders and managers to provide candid responses to our inquiries regarding payment processes, associated barriers, and the timeliness of payments. CHWs and their supervisors were more forthcoming in sharing their perspectives. To ensure an accurate representation of the situation in each district, we spoke with multiple stakeholders involved in the process, allowing us to triangulate the information collected and validate our observations. By choosing two large, distinctly different states and employing a systematic sampling method for districts, blocks, and health facilities, we aimed to provide a comprehensive illustration of the payment processes and the potential barriers and facilitators that may be encountered in diverse settings. Nonetheless, we observed variations between districts within each state, reflecting the well-known phenomenon of significant disparities in health system performance across Indian states and districts. Furthermore, since we selected only two states and two districts per state, our findings may not be generalizable to the entire country. Lastly, the qualitative methodology does not allow us to draw quantitative comparisons between specific characteristics of the payment process, including the partial adoption of digital tools and their effects on payment amounts, timelines, and CHW experiences. Further research, including quantitative data collection, can measure how each step of the process impacts payment timelines, completeness, and CHW motivation, and will furthermore be useful to inform improvement efforts for payment systems

## Conclusions

These findings show that CHW payment processes involve complex sub-processes within the stages of the payment process that can adversely impact payment timelines, CHW workload, and motivation. To address this, policy interventions must include setting clear, simplified, and transparent documentation and timeline standards, and introducing digital tools like payment software, an effective PFMS, and direct bank transfers, which can enhance CHW payment efficiency, performance monitoring, and worker satisfaction. However, the success of these interventions strongly depends on effective fund management across administrative levels and building a positive work culture that prioritizes CHW payments across the payment process steps. Policymakers must bear in mind that improving early payment steps through digital adoption requires strong capacity building of CHWs, especially where their education levels are low, and providing adequate supporting infrastructure is essential. These insights can guide improvements in CHW payment systems in LMICs.

## Supplementary Material

czaf010_Supp

## Data Availability

The data underlying this article cannot be shared publicly for privacy reasons as per the request of the participating organizations.

## Reflexivity statement

Our research team of seven includes six women and one man spanning early, mid-career, and senior researchers. Four members of the team, including the two lead authors, are based in India and three are based in the United States. The authors have a blend of academic, policy, and implementation backgrounds. They have a combined expertise in quantitative and qualitative health systems research in India, the African continent, and the United States, with specializations in health financing, performance-based financing, behavioural economics and its applications to maternal and child health, evidence-based priority setting, quality of care, and public health.
