# Supplementary material for: Community health worker payment processes: a qualitative assessment of experiences in two Indian states
Source: Health Policy Plan. 2025 Feb 15;40(4):483–95. doi: 10.1093/heapol/czaf010 (PMC11979585; doi:10.1093/heapol/czaf010)
Supplement: czaf010_Supp [file czaf010_supp.zip › Supplementary File_clean version.docx]

**Table A1: ASHA activities related to antenatal care, institutional deliveries and child immunizations and their applicable incentive payments**

| **Sr. No.** | **Activities** | **Amount in Rs/case** |
| --- | --- | --- |
| **Common incentives at the national level** | | |
| **Maternal Health** | | |
| 1 | JSY financial package* | |
| a. | For ensuring antenatal care for the woman (at least three ANC checkups including TT injections, IFA tablets) | Rs.300 for Rural areas and Rs. 200 for Urban areas |
| b. | For facilitating institutional delivery | Rs. 300 for Rural areas and Rs. 200 for Urban areas |
| 2. | Reporting Death of women (15-49 years age group) by ASHA to PHC Medical Officer | Rs. 200 for reporting within 24 hours of occurrence of death by phone |
| **Immunization** | | |
| 1 | Full immunization for a child under one year (BCG, OPV 0, 1, 2, 3, Hep B, Pentavalent 1, 2, 3, Rotavirus Vaccine 1, 2, 3, PCV** 1, 2, Measles and Rubella (MR) 1, JE** 1, PCV Booster) | Rs. 100 |
| 2 | Complete immunization per child up-to two years age (all vaccination received between 1st and 2nd year of age after completing full immunization after 01year – MR 2, JE 2, DPT Booster, OPV Booster) | Rs. 75 |
| 3 | Mobilizing children for OPV immunization under Pulse polio Programme | Rs. 100/day |
| 4 | DPT Booster at 5-6 years of age | Rs. 50 |
| **Incentive for Routine Recurrent Activities** | | |
| 1 | Mobilizing and attending VHND or (outreach session/Urban Health and Nutrition Days) | Rs. 200 per session |
| 2 | a) Line listing of households done at beginning of the year and updated every six months  b) Maintaining records as per the desired norms like – village health register  **c) Preparation of due list of children to be immunized updated on monthly basis**  **d) Preparation of due list of ANC beneficiaries to be updated on monthly basis**  e) Preparation of list of eligible couples updated on monthly basis | Rs. 1500 |
| **Additional incentives in Maharashtra** | | |
| **Maternal Health** | | |
| 1 | **Incentive under Pradhan Mantri Matru Vandana Yojana (PMMVY)** |  |
| a. | For registration of pregnant mothers within 12 weeks of pregnancy and filling up form | Rs.100 |
| b. | For completion of all the ANC visits and referring the mother to government hospital for institutional delivery | Rs. 50 |
| c. | After the child receives second Penta vaccination | Rs. 50 |
| 2. | To make a list of pregnant mothers having severe anemia and follow up with them | Rs. 100 |
| **Immunization** | | |
| a. | To report any adverse events after immunization | Rs. 25 |
| b. | To prevent drop-out and actively contribute to achieving aim of complete vaccination of all the children at the village level (for outreach vaccination session) | Rs. 150 |
|  | Incentive under RCH Program Management |  |
| c. | Quarterly meeting for routine immunization | Rs. 75 |
| **Additional incentive in Assam** | | |
| **Maternal Health** | | |
| 1 | Facilitation of High-Risk Pregnancy identification and line listing | Rs. 100 |
| 2 | Follow up of Full ANC with complete routine examination of each pregnant women | Rs. 100 |
| 3 | Mobilizing for screening of HIV of all pregnant women | Rs. 50 |
| 4 | Updating of MCP Card and ensuring opening of bank A/C of beneficiary registered in her area | Rs. 50 |
| **Immunization** | | |
| 1 | Follow-up of full immunization with JE, MR, Rota Virus, Vitamin A, etc. and line listing | Rs. 100 |

*In Low Performing States (LPS) available for all pregnant women delivering in Government Hospitals or accredited private institutions, in High Performing States (HPS) available only for BPL pregnant women, aged 19 years and above. For both LPS and HPS, available for all SC and ST women delivering in a government hospitals or accredited private institutions (<https://nhm.gov.in/WriteReadData/l892s/97827133331523438951.pdf>)

**applicable only in selected districts of few states and in endemic areas

Source: Government of India Ministry of Health And Family Welfare Department Of Health And Family Welfare Lok Sabha Unstarred Question No. 3418 To Be Answered On 13th March, 2020 <http://164.100.24.220/loksabhaquestions/annex/173/AU3418.pdf>

Source: ASHA incentives Master Claim Form, Assam (2019-20) [ASHA_Incentive_Master_Claim_Form_Draft_ENGLISH.pdf (assam.gov.in)](http://nhmssd.assam.gov.in/APPMS/ASHA_documents/ASHA_Incentive_Master_Claim_Form_Draft_ENGLISH.pdf)

**Table A2: List of respondents**

| **In-depth interviews** | | | | | |
| --- | --- | --- | --- | --- | --- |
| **State** | **Assam** | | **Maharashtra** | | **Total** |
|  | State Community Mobilizer | | Program Manager-ASHA | | 8 |
|  | State Finance Manager | | State Finance Administrator | |  |
|  | State Program Manager | | State Program Manager | |  |
|  | Programmer (IT) | | State IT Team | |  |
| **District** | **District 1 (performance quartile 2)** | **District 2 (performance quartile 2)** | **District 1**  **(performance quartile 2)** | **District 2 (performance quartile 3)** | 9 |
|  | District Accounts Manager | District Accounts Manager | District Accounts Manager | District Accounts Manager |  |
|  | District Community Mobilizer | District Community Mobilizer | District Community Mobilizer | District Community Mobilizer |  |
|  |  |  | District Program Manager |  |  |
| **Block** | Block Community Mobilizer | Block Community Mobilizer | Block Community Mobilizer | Block Community Mobilizer | 15 |
|  | Block Accounts Manager | Block Accounts Manager | Block Accounts Manager | Block Accounts Manager |  |
|  | Additional Block Program Manager (2) | Block Program Manager | Block Health officer | Block Health officer |  |
|  |  | Block Health Officer |  |  |  |
|  |  | Additional Block Program Manager |  |  |  |
| **Primary Health Centre** | ASHA (2) | ANM (2) | ASHA (2) | ASHA (1) | 21 |
|  | Block Facilitator (ASHA Supervisor- 2) |  | Block Facilitator (ASHA Supervisor- 2) | Block Facilitator (ASHA Supervisor- 2) |  |
|  | ANM (2) |  | ANM (2) | ANM (2) |  |
|  |  |  | Medical Officer in-charge (PHC) | Medical Officer in-charge (PHC) |  |
| **Total number of interviews conducted** | | | | | **53** |
| **Focus Group Discussions** | | | | | |
| With ASHAs | | | | | 4 focus groups  (8 members per FGD) |
| With Block Facilitator/ ASHA Supervisor (8 members per FGD) | | | | | 4 (8 members per FGD) |
| **Total number of FGD conducted** | | | | | **8** |

**Table A3: List of codes, sub-codes and themes**

| **Codes** | **Sub-codes*** | **Themes & sub-themes** |
| --- | --- | --- |
| Recording work and Submitting Claims | *Efficiency*  Accountability  Transparency  *Motivation* | - Excessive documentation with duplication where policies are unavailable, or stakeholders are unclear on documentation requirements - Delays in submitting forms and approvals due to unclear policies on documentation - Increased efficiency is possible through the digitization of records, however, involve adoption challenges for ASHAs and their supervisors - Increased workload for ASHAs to maintain excessive/duplicative documentation for obtaining payments - Out-of-pocket costs are associated with documentation required for claiming incentives |
| Verification of Claims | *Efficiency*  *Accountability*  Transparency  *Motivation* | - Verification based on review of signed paper records with a fear of future financial audits - Burden of obtaining multiple administrative signatures on ASHAs leads to payment delays - Local initiatives to streamline documentation ease the verification process |
| Processing and Approval of claims | *Efficiency*  *Accountability*  *Transparency*  *Motivation* | - Manual claim processing is prone to errors which is stressful for managers - Digital claim forms ease the approval process - Teams that prioritize ASHA payments find ways to ensure timely processing of claims |
| Disbursement and Mode of Payment | *Efficiency*  *Accountability*  *Transparency*  *Motivation* | - The Public Financial Management System simplified integrated payments - ASHAs prefer direct bank transfers to earlier modes of payment |
| Accessing Payments | *Efficiency*  *Accountability*  *Transparency*  *Motivation* | - Quicker access through bank transfers - ASHAs informed of payment receipt through SMS - Decrease in corruption and increase in accuracy of payments - Liquidity of funds at the local administrative levels determines timely payments |
| Supervision, Monitoring and Review | *Efficiency*  *Accountability*  *Transparency*  *Motivation* | - Digital payment software enables efficient monitoring of ASHA performance - Performance reports serve as supportive supervisory tools for recognition and punitive actions - Payment timelines can be monitored through payment software |

**Sub-codes in italics denote those for which the themes identified in the data could be mapped.*
